# Carotenoids in the Context of the Mediterranean Dietary Pattern: From Food Matrix and Bioavailability to Biomarkers and Human Health

**DOI:** 10.3390/antiox15091164

**Published:** 2026-09-14

**Authors:** Giuseppina Augimeri, Mary Fucile, Alberto Vaccarella, Giancarlo Statti, Filomena Conforti, Daniela Bonofiglio

**Affiliations:** 1 Department of Pharmacy and Health and Nutritional Sciences, University of Calabria, Via P. Bucci, Arcavacata di Rende, 87036 Cosenza, Italy; giuseppina.augimeri@unical.it (G.A.); mary.fucile@unical.it (M.F.);; 2 Centro Sanitario, University of Calabria, Arcavacata di Rende, 87036 Cosenza, Italy

**Keywords:** mediterranean diet, carotenoids, plant food matrix, xanthophylls, bioaccessibility, skin carotenoids, Veggie Meter^®^

## Abstract

The Mediterranean Diet pattern is characterized by high intake of plant-based foods rich in bioactive nutrients, including carotenoids. This review examines the major dietary carotenoids and xanthophylls characteristic of the Mediterranean food matrix, focusing on their structural diversity, plant biosynthesis, release from the food matrix, bioavailability, and biological redox-modulating activities. The literature was collected from PubMed, Web of Science, and Scopus to trace the pathway of carotenoids from their accumulation in plant tissues to their potential effects on human health. Culinary processing and the concomitant intake of dietary lipids can enhance carotenoid bioaccessibility by promoting isomerization and facilitating the formation of mixed micelles during digestion. In addition to systemic absorption, noninvasive diagnostic tools, including skin carotenoid measurements by reflectance spectroscopy and macular pigment optical density, provide useful biomarkers of fruit and vegetable intake. Carotenoids may contribute to systemic antioxidant and redox regulation by quenching singlet oxygen, scavenging free radicals, and modulating redox-sensitive signaling pathways. Overall, their structural diversity, food-matrix interactions, bioaccessibility, and biological activities support continued investigation of carotenoid-rich foods within the Mediterranean dietary pattern and their associations with human health outcomes.

## 1. Introduction

The health and well-being of humans are influenced by a multitude of factors, including lifestyle choices involving a healthy and balanced diet and daily physical activity. The consumption of ultra-processed foods, which characterize the Western Diet, is associated with the development of non-communicable chronic diseases, including obesity, metabolic syndrome, gastrointestinal diseases, cardiovascular disease, and some types of cancer [1]. Unlike the Western Diet, the Mediterranean Diet (MD) is one of the most effective for promoting health [1]. Indeed, high adherence to this dietary model has been found to improve the prognosis and delay the onset of various pathologies including visual ones [2], as well as improving cognitive functions [3]. The MD recommends a high intake of plant-origin foods, especially fruits and vegetables, along with wholemeal products, pulses and fish, and a limited intake of red meat and sweets [4]. Fruits and vegetables are rich in several bioactive compounds, including carotenoids, lipophilic natural pigments belonging to the terpenoid family. Although they are characterized by wide structural diversity and numerous physicochemical and biological properties, based on the presence of oxygen atoms, they can be structurally divided into two classes, carotenes and xanthophylls [5]. About 700 carotenoids occur in nature and are produced by plants, algae, fungi and microorganisms [6]. However, only 50 are consumed through the diet, and a small fraction of those, including α-carotene, β-carotene, β-cryptoxanthin, lutein, lycopene and zeaxanthin, are found in human tissue or blood [7]. Carotenoids show different beneficial effects, since some of them have provitamin activity, whereas others have photoprotective and antioxidant functions [8,9]. Within biological systems, they act as non-enzymatic antioxidants through two main mechanisms: physical quenching of singlet oxygen (O_2_) and chemical neutralization of reactive oxygen and nitrogen species (ROS/RNS), such as superoxide anions, hydroxyl radicals, and peroxyl radicals. By acting as antioxidants capable of interrupting the reaction chain, carotenoids intercept reactive oxygen and nitrogen species (ROS/RNS) before they can trigger a cascade of oxidative damage to vital cellular components, including membrane phospholipids, structural proteins, and nucleic acids [10].

Although the health-related effects of the MD cannot be attributed to a single bioactive compound but rather arise from the interactions of multiple dietary components, including unsaturated fatty acids, dietary fiber, vitamins, minerals, polyphenols and other phytochemicals, several physicochemical strategies are being developed to optimize carotenoid extraction from plants and the food matrix, as well as their biochemical characterization. Their investigation within the Mediterranean dietary pattern is nevertheless relevant for several reasons. First, carotenoid-rich fruits and vegetables are prominent components of this dietary pattern. Second, carotenoid bioaccessibility is strongly influenced by the food matrix and by co-ingested lipids; therefore, the habitual combination of plant foods with EVOO and traditional culinary preparations such as tomato-based sofrito provides a relevant dietary context in which carotenoid release, isomerization and micellization can be examined. Third, circulating and tissue carotenoid concentrations provide objective measures of carotenoid exposure and have been associated with fruit and vegetable intake and with indices of MD adherence, although they are not specific biomarkers of the MD. This review aims to provide an up-to-date overview of the carotenoids found in plant-based foods consumed in the Mediterranean region, ranging from their phytochemical diversity and stability to their bioaccessibility and bioavailability in humans, with a particular focus on their antioxidant and redox-modulating properties against reactive oxygen species (ROS). Special attention will be given to the mechanisms underlying their antiradical activity and to recently characterized carotenoids or newly described antioxidant functions. Furthermore, we will discuss the use of plasma, skin, and macular carotenoids as biomarkers of health, as well as their emerging role as objective indicators of fruit and vegetable intake and adherence to the Mediterranean diet. Of note, the reference to the Mediterranean dietary pattern in this review defines the dietary and food-matrix context in which carotenoids are examined, rather than implying that carotenoids are unique to, or solely responsible for, the effects associated with the MD.

## 2. Search Strategy and Data Extraction

This narrative review summarizes the current literature on carotenoids found in Mediterranean plant-based foods, focusing on carotenes and xanthophylls. An exhaustive literature search was conducted on PubMed, Web of Science Core Collection, and Scopus for records published between January 2000 and June 2026, using the following Boolean search string, applied consistently across all three databases: (“carotenoids” OR “carotenes” OR “xanthophylls” OR “lycopene” OR “lutein” OR “beta-carotene”) AND (“Mediterranean diet” OR “Mediterranean food*”) AND (“bioavailability” OR “bioaccessibility” OR “biomarkers” OR “health”). The search retrieved 726 records. After removal of duplicates, titles and abstracts were independently screened against pre-defined eligibility criteria: full-text, peer-reviewed, English-language original studies (in vitro, in vivo, and human clinical trials) and mechanistic reviews addressing carotenoid chemistry, plant biosynthesis, food-matrix interactions, human bioavailability, or biological activity relevant to the Mediterranean dietary pattern. Conference abstracts, book chapters, non-peer-reviewed sources, non-English-language articles, and studies without a clear methodological link to carotenoid chemistry or Mediterranean food matrices were excluded. This process yielded 162 articles for inclusion. A limited number of additional foundational references on carotenoid metabolic pathways and analytical methodology, identified through backward citation searching, were incorporated to provide mechanistic context where needed.

The collected material was organized into the following sections: (I) structural diversity, biosynthesis, and accumulation of carotenoids in edible plants as a function of ripening; (II) major carotenoid-rich foods in the Mediterranean diet; (III) stability, analytical profiling, and bioavailability of dietary carotenoids; (IV) assessment of carotenoid status in humans through plasma, skin, and macular biomarkers; and (V) biological activities of carotenoids relevant to human health, including antioxidant, anti-inflammatory, cardiometabolic, ocular, and cancer-related effects. Finally, the role of carotenoids as objective biomarkers of fruit and vegetable intake and adherence to the Mediterranean diet is highlighted.

## 3. Dietary Carotenoids in Mediterranean Plant Foods

### 3.1. Structural Diversity and Classification

**Figure 1:**
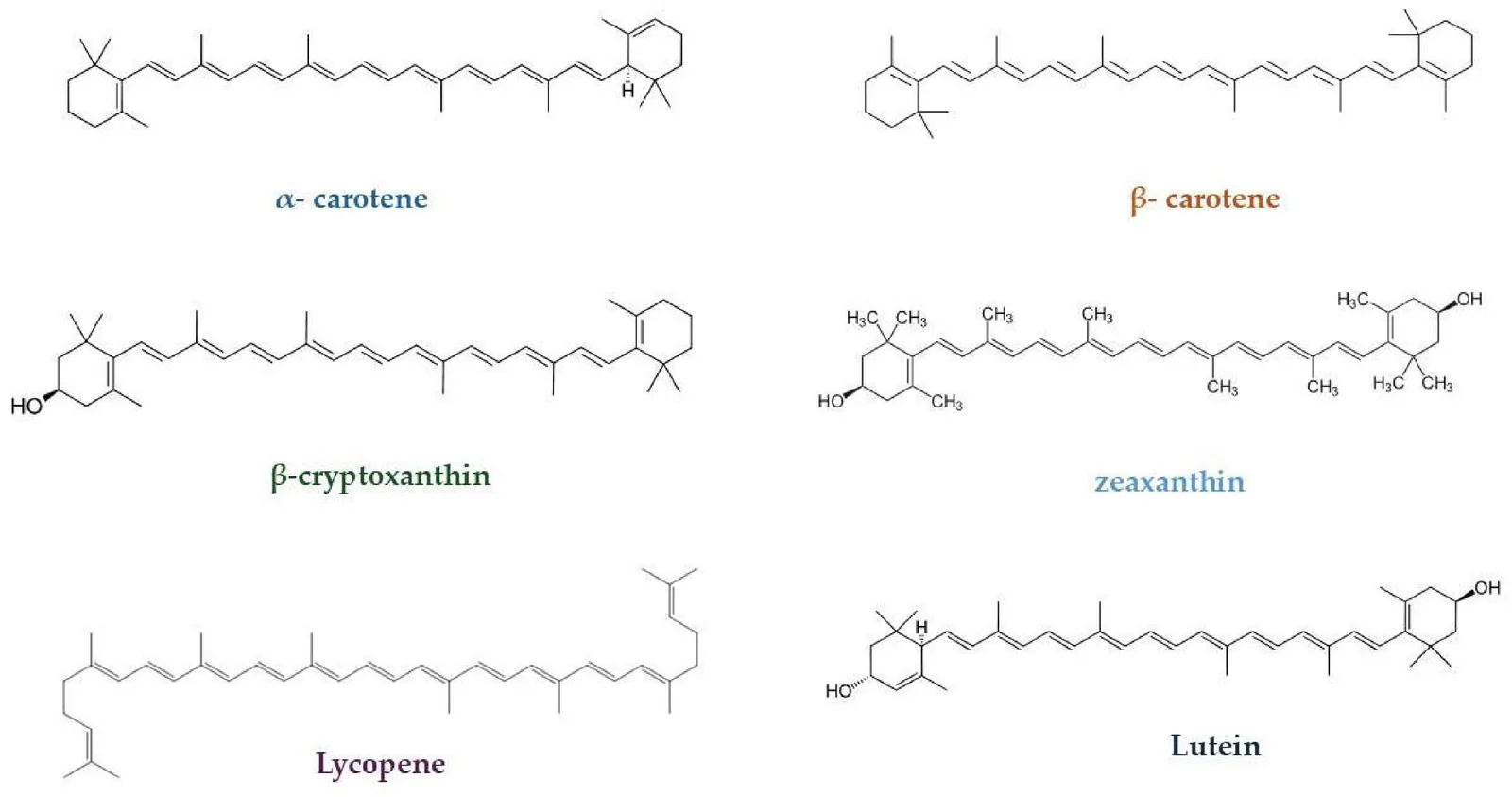
Chemical structure of different carotenoids.

Carotenoids are pigments synthesized by a wide spectrum of photosynthetic and non-photosynthetic organisms, including prokaryotes, fungi, algae and plants [6,11]. More than 750 carotenoids exist in nature [12]. From a phytochemical standpoint, these compounds belong to the terpenoid family; specifically, they are tetraterpenes (C40) and are derived from the condensation of eight isoprene units (C5) originating from the metabolic pathways of mevalonic acid (MVA) or 2-C-methyl-D-erythritol-4-phosphate (MEP). To date, more than 750 naturally occurring carotenoids have been identified [12]. From a chemical standpoint, their structure is characterized by a central polyene chain containing a variable number of conjugated double bonds. This structure represents the molecule’s chromophore, which is responsible for its potent antioxidant activity and determines their distinctive coloration, ranging from yellow to orange and deep red [13,14,15,16]. Based on their structural characteristics, carotenoids are traditionally divided into two main categories: carotenes, which are purely hydrophobic and nonpolar hydrocarbons containing no oxygen atoms in their structure; and xanthophylls, oxygenated derivatives of carotenes, which are more polar due to the presence of oxygen-containing functional groups, such as hydroxyl (OH), epoxide (O-), ketone (O), or carboxyl groups. For the former, representative examples include alpha-carotene, beta-carotene, and lycopene, while for the latter, representative examples include lutein, zeaxanthin, and beta-cryptoxanthin (Figure 1). Furthermore, within these two main classes, carotenoids can be subdivided into subclasses based on their acyclic or cyclic nature. Acyclic carotenoids, such as lycopene, have linear structures, while cyclic carotenoids contain ionone rings at one or both ends of the polyene chain, such as beta-carotene or lutein. In senescing plant tissues, fruits, and flowers, hydroxylated xanthophylls are often esterified with fatty acids, which increases their stability and lipophilicity within cellular chromoplasts [15]. Due to this highly hydrophobic nature, all carotenoids are insoluble in water but highly soluble in nonpolar organic solvents and lipids [16].

### 3.2. Biosynthetic Origin of Carotenoids in Edible Plants and Ripening-Dependent Accumulation

**Figure 2:**
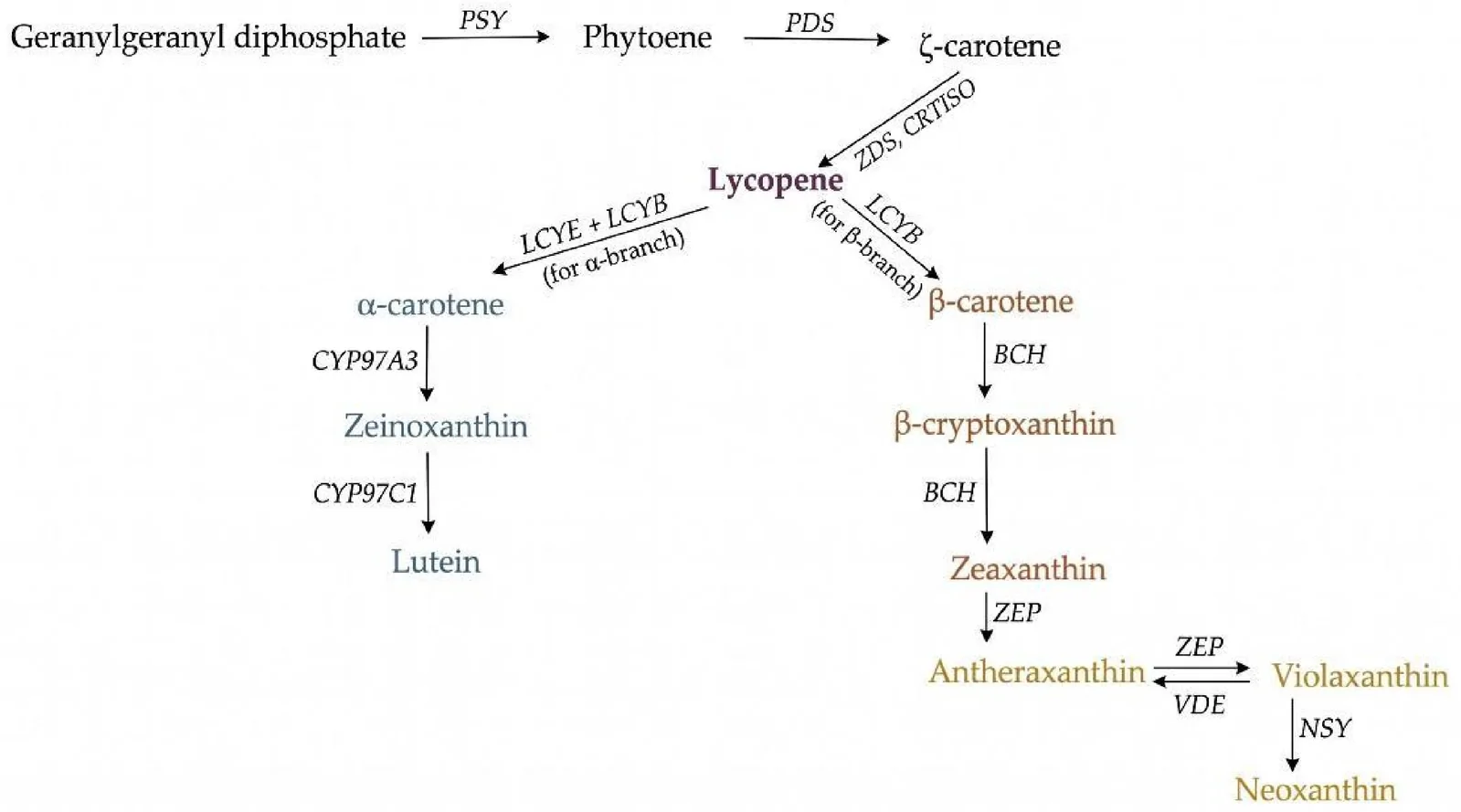
Biosynthetic pathways of carotenoids: geranylgeranyl diphosphate is sequentially converted into phytoene-by-phytoene synthase (PSY), then to ζ-carotene by phytoene desaturase (PDS), and subsequently to lycopene via ζ-carotene desaturase (ZDS) and carotenoid isomerase (CRTISO). Lycopene serves as a central branching point: it enters the ε,β-branch via lycopene ε-cyclase (LCYE) and lycopene β-cyclase (LCYB) to yield α-carotene, which is converted to lutein by cytochrome P450-dependent hydroxylases (CYP97A3 and CYP97C1) via zeinoxanthin. Alternatively, LCYB directs lycopene into the β,β-branch to form β-carotene, which is hydroxylated by β-carotene hydroxylase (BCH) to β-cryptoxanthin and zeaxanthin. Finally, zeaxanthin is reversibly converted to antheraxanthin and violaxanthin by zeaxanthin epoxidase (ZEP) and violaxanthin de-epoxidase (VDE), and ultimately to neoxanthin by neoxanthin synthase (NSY).

Carotenoids are synthesized in the chloroplasts from isopentenyl pyrophosphate, which is transformed into geranylgeranyl diphosphate (GGPP) through the methylerythritol-4-phosphate pathway [17]. Next, two molecules of GGPP are condensed into phytoene by phytoene synthase (PYS), which, through several reactions catalyzed by phytoene desaturase (PDS), ζ-carotene desaturase (ZDS), and at least two isomerases, including carotenoid isomerase (CRTISO), is desaturated to lycopene. This compound can follow two different pathways, the ε, β, or the β branch, through which it can be further cyclized by lycopene β-cyclase (LCYB) and lycopene ε-cyclase (LCYE) to form α-carotene or β-carotene, respectively. α-carotene can be converted into lutein through the activity of two distinct carotenoid hydroxylases belonging to the cytochrome P450 family, β-ring hydroxylase (CYP450-BCH) and ε-ring hydroxylase (CYP450-ECH). In the β branch of the carotenoid pathway, hydroxylation of β-carotene by β-carotene hydroxylase (BCH) produces zeaxanthin via β-cryptoxanthin for the xanthophyll cycle. Epoxidation of zeaxanthin in the ABA biosynthetic pathway leads to the formation of violaxanthin and neoxanthin [18]. In addition to phytoene synthase (PSY), the plant Orange (Or) protein is also important for carotenoid biosynthesis and accumulation [19], as it functions as a holdase chaperone that post-transcriptionally regulates phytoene synthase (PSY) [20] (Figure 2).

Carotenoids have different functions in plants. They are involved in the process of photosynthesis, contributing to the light-harvesting process and protecting the plant from photooxidation [11]. In particular, they absorb blue-green light (450–550 nm) and transfer the captured energy to the chlorophyll within the light-harvesting antenna complex [21]. Upon exposure to excess light, the antenna complex associated with photosystem II in plants dissipates the excess of energy through a series of photoprotection mechanisms known as non-photochemical quenching (NPQ) [22,23,24]. This process is regulated through the xanthophyll cycle, which involves the de-epoxidation of violaxanthin to zeaxanthin in a reversible reaction. Indeed, it has been observed that violaxanthin accumulates under moderate light conditions, while zeaxanthin predominates when plants are exposed to high light intensity [25]. Facilitating energy dissipation, carotenoids contribute to the detoxification of reactive oxygen species [22,23]. Apart from their role in photosynthesis, carotenoids are precursors of some phytohormones, especially strigolactones (SL), abscisic acid (ABA) (19,25) and volatile compounds [11]. Moreover, they influence plant development and confer characteristic color and nutritional value to fruits [11]. Finally, by accumulating in the ripe fruits and flowers, they attract animals for seed dispersal and reproductive purposes [11]. Dietary intake of carotenoids is strongly influenced by the stage of ripeness of food sources at the time of consumption. As fruits and vegetables ripen, their internal cellular structures undergo a transformation that converts green, photosynthetic chloroplasts into pigmented chromoplasts capable of storing nutrients. Fruit ripening is a continuous and highly coordinated process in which ROS, primarily hydrogen peroxide (H_2_O_2_), act as endogenous signaling molecules that help trigger chlorophyll breakdown and the onset of pigment biosynthesis. Carotenoids, as established non-enzymatic antioxidants, are well positioned to help buffer this internal oxidative environment by physically quenching singlet oxygen and scavenging free radicals; however, whether their ripening-associated accumulation is itself a direct antioxidant response to ROS signaling, as opposed to a downstream consequence of the broader ripening program, remains to be firmly established [26]. This specific stage of ripeness determines both the total amount of carotenoids and their chemical quality, physical state, and resulting bioavailability in the human body. In tomatoes (*Solanum lycopersicum*), the transition from the immature green stage to the fully ripe red stage significantly alters the carotenoid profile. In unripe or partially ripe tomatoes, the total carotenoid content is relatively low and is dominated by polar xanthophylls such as lutein. As the fruit reaches full red maturity, there is an exponential increase in lycopene. However, at this optimal stage for consumption, lycopene aggregates into dense, rigid microcrystalline structures within the cell membranes [27]. In olive (*Olea europaea*) fruit, the main carotenoids are lutein and 9-cis-neoxanthin. Unlike tomatoes or peppers, these xanthophylls exhibit high baseline stability during the early and intermediate stages of ripening, resisting degradation until the fruit is significantly overripe. The transfer of xanthophylls such as lutein and 9-cis-neoxanthin from the olive pulp into the oil during processing is influenced primarily by the water content of the pericarp, and only to a lesser extent by the ripening stage itself. According to Vicario et al., olive pastes with higher water content are more fluid and undergo less mechanical tissue disruption during hammer-crushing, resulting in lower pigment extraction into the oil; conversely, pastes derived from lower-moisture pulp are more effectively disrupted, releasing more xanthophylls into the oil phase. This effect was more pronounced for xanthophylls (lutein, 9-cis-neoxanthin) than for chlorophylls. Since agronomic factors such as irrigation and soil management strongly affect pericarp water content, they may therefore represent an additional, indirect lever on the carotenoid richness of the final oil, alongside fruit ripeness [28].

### 3.3. Main Carotenoid-Rich Foods in the Mediterranean Diet

Fruits and vegetables represent the main dietary source of carotenoids. Generally, increasing fruit and vegetable ripeness increases carotenoid concentration [18,29]. However, the final carotenoid content is greatly influenced by genetic characteristics and agroecological conditions of fruits and vegetables [21,29,30]. Root vegetables are excellent sources of α- and β-carotene [31], whereas tomato is the main source of lycopene [32]; berries such as blueberries and green leafy vegetables in general are good sources of several carotenoids, including lutein and β-carotene [18,33]; corn kernels represent one of the main sources of lutein and zeaxanthin [34]; mango is a good source of β-carotene, but also of lutein [35]; another good source of β-carotene is sweet pepper [36]; phytoene (PT) and phytofluene (PTF) are present in many foods, including carrot, apricot, tomato sauce and juice, orange [37]; citrus fruits are rich in xanthophylls, including β-cryptoxanthin present in mandarin [38,39].

### 3.4. Food Matrix Interactions and Dietary Synergies: Interactions with Lipids, Polyphenols and Dietary Fiber

The bioavailability and biological activity of carotenoids in the Mediterranean diet depend strongly on the composition of the entire meal. Within the digestive system, the simultaneous presence of lipids, polyphenols, vitamins, and fiber alters the chemical stability, micellization rates, and metabolism of these pigments [40,41]. Dietary fats play a key role in the absorption of these lipophilic compounds. The simultaneous consumption of carotenoids with extra virgin olive oil (EVOO) facilitates their transfer into the aqueous micellar phase during duodenal digestion, which is an essential prerequisite for their absorption by enterocytes [41,42]. Oils rich in unsaturated fatty acids (particularly monounsaturated fatty acids, MUFAs, and polyunsaturated fatty acids, PUFAs) promote greater micellization compared to saturated fats. Specifically, oleic acid—the predominant fatty acid in EVOO—facilitates the assembly and basolateral secretion of carotenoid-rich chylomicrons [41,43]. However, the degree of micellization is finely modulated by the underlying food matrix and the polarity of the specific molecule: xanthophylls, which possess oxygenated functional groups, consistently exhibit greater bioaccessibility and micellization efficiency than nonpolar carotenoids [41,44]. Traditional Mediterranean culinary practices further enhance these matrix synergies. “Sofritto,” a fundamental culinary preparation that combines tomatoes, onions, garlic, and extra virgin olive oil, combines gentle heat treatment with the presence of unsaturated lipids. This process optimizes both the physical release of lycopene from chromoplast structures and its geometric isomerization from the native *all-E* (*trans*) configuration to *Z* (*cis*) isomers, which exhibit greater bioaccessibility, solubility, and bioactivity [45,46]. The addition of specific aromatic ingredients rich in organosulfur compounds during tomato cooking synergistically promotes the isomerization of lycopene into Z isomers, thereby maximizing its incorporation into mixed micelles [41,45]. Furthermore, the concurrent consumption of olive oil directly modulates the absorption of carotenoids from tomato matrices, while favorably attenuating postprandial lipemia [42]. In addition to lipids, the polyphenols and vitamins present in the Mediterranean diet form an integrated redox network that protects carotenoids from oxidative degradation during gastrointestinal digestion and subsequent systemic transport [40,47]. The phenolic compounds found in abundance in extra virgin olive oil (such as hydroxytyrosol and oleuropein) and dietary flavonoids (e.g., quercetin and catechins) directly scavenge free radicals, inhibiting lipid peroxidation and neutralizing reactive oxygen species (ROS) within the digestive tract [40]. Plant terpenoids act as endogenous lipophilic antioxidants within the dietary phytocomplex, providing mutual protection against autoxidation [47,48]. Furthermore, it has been demonstrated that a new combined biomarker, which integrates plasma carotenoids, vitamin C, and ferric reduction antioxidant power (FRAP), shows a stronger correlation with fruit and vegetable intake than any of the individual components considered in isolation [49]. At the lipid-water interface of the micellar mixture, dietary phenolic compounds and ascorbic acid can directly reduce and regenerate oxidized carotenoid radicals, thereby prolonging their chemical stability and physiological shelf life. This cooperative “protective effect” significantly attenuates the autoxidation and epoxidation of carotenoids, preserving their native conjugated polyene structure and their ability to scavenge free radicals [40,47]. Furthermore, the antioxidant action of dietary polyphenols occurs both locally within the gastrointestinal lumen and systemically, where they modulate the systemic redox state and inflammatory signaling cascades [40,47]. The plant cell wall matrix and dietary fibers act as a physical barrier that regulates the controlled release of carotenoids during gastrointestinal transit. The structure of the matrix (which includes the morphology of chromoplasts, cell wall rigidity, and pectin content) significantly modulates bioavailability: indigestible polysaccharides can limit micellization through physical entrapment and increased luminal viscosity [40,41]. Conversely, home-cooking techniques, which alter the cellular microstructure, promote the release of pigments, as clearly demonstrated in the case of leafy green vegetables such as spinach, where thermal and mechanical treatments compromise tissue integrity and promote the release of carotenoids [50]. The gut microbiota complements this metabolic network by converting carotenoids and polyphenols into small bioactive metabolites. Carotenoids interact dynamically with the gut microbiota, undergoing enzymatic cleavage that leads to the production of apocarotenoids, which reduce inflammation of the intestinal mucosa and preserve the integrity of the epithelial barrier [51]. Furthermore, adherence to the Mediterranean diet promotes the growth of beneficial bacteria that produce short-chain fatty acids (SCFAs), such as *Faecalibacterium*, *Roseburia*, and *Bifidobacterium*, which in turn modulate intestinal absorption, local bioactivity, and the immunological responses triggered by carotenoids and polyphenols [52,53]. At the same time, the phenolic compounds present in olive oil undergo biotransformation in the colon, generating low-molecular-weight phenolic metabolites capable of modulating both epithelial signaling and the microbial ecosystem (Table 1) [52].

**Table 1:** Redox mechanisms and protective functions of major carotenoids within Mediterranean food matrices.

Matrix Type	Mediterranean Examples	Major Carotenoids	Main Antioxidant Mechanism	References
Fruits and vegetables	Tomato, pepper	Lycopene, β-carotene	Singlet oxygen quenching and free radical scavenging during ripening-associated oxidative stress	[18,36,37]
Leafy vegetables	Spinach, chard	Lutein, β-carotene, zeaxanthin	Photoprotection via the xanthophyll cycle, non-photochemical quenching (NPQ), and ROS scavenging.	[18,33]
Root vegetables	Carrot	α-carotene, β-carotene	Radical scavenging and protection against oxidative damage in storage organs.	[31,36]
Citrus fruits	Orange, mandarin	β-cryptoxanthin, lutein, violaxanthin	ROS scavenging and oxidative stability through esterified xanthophyll accumulation	[37,38,39]
Berries	Blueberry, bilberry	Lutein, β-carotene	Free-radical scavenging supporting fruit development and ripening	[18,33]
Cereal grains	Corn kernels	Lutein, zeaxanthin	Photoprotection through xanthophyll-mediated energy dissipation and ROS quenching	[34]
Lipid matrices	Olive, EVOO (extra virgin olive oil)	Lutein, 9-cis-neoxanthin, β-carotene	Radical scavenging and inhibition of lipid peroxidation during oil processing and storage	[28,39]

## 4. Stability, Analytical Profiling and Bioaccessibility of Dietary Carotenoids

### 4.1. Stability, Isomerization and Degradation

In Mediterranean plant foods, carotenoids are predominantly present in the thermodynamically stable trans configuration, often incorporated into crystalline substructures within chromoplasts, giving them relative resistance to thermal damage. However, processing methods such as cooking or dehydration alter this native state, triggering a geometric isomerization from trans to cis (E/Z). This is an interesting phenomenon from a nutritional standpoint: while all-trans isomers exhibit low solubility and high crystallinity, Z isomers possess altered physical properties that enhance their absorption into mixed micelles during digestion. Consequently, Z isomers exhibit significantly higher bioavailability and accumulation efficiency in human tissues compared to their trans counterparts [40,41]. It is interesting to note that, in traditional Mediterranean culinary matrices, this isomerization is synergistically enhanced. The co-presence of dietary lipids such as olive oil, along with specific ingredients from the genera Allium and Brassica, which are rich in organosulfur compounds such as polysulfides and electrophilic isothiocyanates, acts as a catalytic system that promotes thermal isomerization to the Z form without inducing pigment decomposition [40,42].

Xanthophylls often undergo esterification with fatty acids during plant maturation. This structural change reduces their polarity, directly affecting their digestive stability [41]. At the same time, the highly unsaturated polyene chain is responsible for the carotenoids’ antioxidant properties and makes them highly susceptible to irreversible oxidative degradation. This autoxidative process follows first-order kinetics and is accelerated by high temperatures, light exposure, and the presence of molecular oxygen [43,44,45]. Peroxyl radicals are trapped by addition to the conjugated system, forming resonance-stabilized radicals centered on carbon [40,44]. The initial stages of non-enzymatic oxidation involve epoxidation and the subsequent eccentric or central cleavage of the polyene chain [44,45]. This degradation pathway produces a homologous series of non-volatile apo-carotenoids—such as β-apo-carotenals and β-apo-carotenones (including retinal)—along with low-molecular-weight volatile aromatic compounds (e.g., 6-methyl-5-hepten-2-one and citral), which serve as chemical markers of quality loss [40,44].

### 4.2. Analytical Profiling

The accurate in vitro identification of carotenoids from different matrices constitutes a challenge due to various factors, including structural heterogeneity, the susceptibility of these molecules to isomerization and oxidation, and the differences in solubility and bioaccessibility during digestion. After sample collection and botanical authentication of the plant, a cleaning phase to remove traces of soil or any contaminants is required. Then, the plant tissue is fragmented into small particles (homogenization phase), before the extraction step is carried out. The extraction of carotenoids can be performed by classical approaches, such as maceration, or innovative and green extraction techniques, including ultrasound-assisted extraction and microwave-assisted extraction, which allow solvent and time savings [46]. Another extractive technique is represented by high hydrostatic pressure, which is a technology that does not use heat to extract secondary metabolites, such as carotenoids [47]. After the extraction phase, the carotenoids undergo purification and concentration phases, followed by the identification and chemical analysis phases [47]. A technique utilized to identify the presence of carotenoids is mass spectrometry (MS), which, operating in positive and negative electrospray ionization (ESI) mode, acts by ionizing molecules. Since only xanthophylls ionize with high efficiency, most carotenoids can be identified through other techniques, including the high-performance or ultra-high-performance liquid chromatography (HPLC and UHPLC) with a diode array detector. Moreover, to characterize the total carotenoid content, combined techniques, such as UHPLC-ESI-MS/MS [48] or liquid chromatography-photodiode array-mass spectrometry/quadrupole time-of-flight (LC-PDA-MS/QTof), are applied [49]. Regarding LC technology, HPLC can be coupled with a diode array detector (DAD) detector for quantification and identification of carotenoids, allowing for the analysis of the ultraviolet-visible (UV-Vis) absorption spectrum covering the spectrum from 220 nm to 700 nm. Another technique utilized for the identification and quantification of carotenoids is the coupled LC-MS/MS technique with atmospheric pressure chemical ionization (APCI+), a more advanced method, which allows safer identification and more accurate quantification of carotenoids [47].

### 4.3. Food Processing, Matrix Release

In their natural, raw state, the rigidity of cell walls and organelle membranes prevents digestive enzymes from acting effectively. Furthermore, the efficiency of release from the matrix is linked to the physical form in which the pigment is deposited and to the morphology of the organelles: in green leafy vegetables, carotenoids are strongly bound to protein complexes in the chloroplasts, resulting in markedly reduced bioaccessibility; in non-photosynthetic tissues, however, they are found in chromoplasts in the form of compact, solid crystalline aggregates (such as lycopene in tomatoes or carotenes in carrots) or dissolved in lipid droplets and crystalline structures, with the latter forms being inherently more bioaccessible and easier to solubilize [50,51,52]. Conventional heat treatments, such as boiling, pressure cooking, blanching, and dehydration, alter this native state, acting as promoters of bioaccessibility. The heat denatures protein-carotenoid complexes, breaks down organelle membranes, and induces the depolymerization and solubilization of cell wall constituents. In particular, the increase in temperature accelerates the β-elimination processes of pectins, causing plant tissues to soften and increasing the fraction of pigment that is extractable and solubilizable during digestion. This positive effect more than compensates for the partial losses of total carotenoids due to oxidative thermal degradation. Exposure to heat induces a dose- and time-dependent geometric isomerization from the native all-trans configuration to the cis configuration. From a nutritional standpoint, cis isomers are less prone to crystallization and aggregation, have a shorter molecular length, and exhibit greater polarity, physicochemical characteristics that facilitate their release from the matrix and maximize their efficiency of incorporation into mixed intestinal micelles compared to trans forms [50,51]. Mechanical treatments, such as homogenization or grinding, reduce the particle size of the plant material, thereby increasing the surface area exposed to enzymatic action and synergistically enhancing the effect of heat. However, interaction with other components of the matrix can alter absorption; for example, the presence of dietary fiber can trap carotenoids within the fibrous network and increase the macroviscosity of gastric fluids, which reduces peristaltic mixing and hinders the adequate diffusion of lipases and bile salts [50]. Furthermore, the chemical state of carotenoids in the plant matrix significantly influences their behavior during digestion. Xanthophylls often undergo esterification with fatty acids during plant maturation. This structural change reduces their polarity and directly affects their bioaccessibility; free xanthophylls generally exhibit greater micellization and higher release from the food matrix compared to mono- or di-esterified forms, which are more hydrophobic in digestive fluids [41]. However, the ultimate bioavailability remains strictly dependent on the presence of a lipid phase consumed as part of the same meal or used as a cooking medium. Dietary lipids provide the necessary hydrophobic environment in which the released carotenoids can dissolve. Fat intake also stimulates the secretion of bile salts and pancreatic lipases, supporting the formation of the mixed micelles necessary for transport to the enterocytes. At this stage, the nature of the lipids determines the efficiency of the process: oils rich in long-chain and unsaturated fatty acids (typical of the Mediterranean diet) increase the solubilizing capacity of the micelles compared to medium-chain or saturated fatty acids [50].

### 4.4. Bioaccessibility and Human Metabolism

In their natural, raw state, the rigidity of cell walls and organelle membranes prevents digestive enzymes from acting effectively. Furthermore, the efficiency of release from the matrix is linked to the physical form in which the pigment is deposited and to organelle morphology. Most animals, including humans, are unable to synthesize carotenoids and need to obtain them from dietary sources such as fruits and vegetables [17]. After the ingestion of carotenoid-enriched foods, the initial phase of carotenoid release from the food matrix occurs through mechanical fragmentation during chewing and chemical digestion by the salivary enzymes, followed by gastric and intestinal digestion. Not all carotenoids released from the food matrix during digestion are ultimately absorbed by enterocytes: a considerable fraction remains unincorporated into mixed micelles or fails to cross the intestinal brush border and is instead excreted in the feces rather than entering the systemic circulation [53]. This upper limit of intestinal absorption becomes particularly evident following high consumption of carotenoid-rich vegetables, where it has been shown that fecal excretion of carotenoids increases in proportion to dietary intake, confirming that a substantial portion of the ingested pigments passes through the gastrointestinal tract without being absorbed [54]. However, it has been shown that various practical strategies can shift this balance toward absorption rather than excretion. Mechanical processing (e.g., chopping, blending, homogenization) and heat treatment break down plant cell walls and release carotenoids from the chromoplast matrix, increasing their bioaccessibility several-fold compared to raw vegetables [55]. Consuming vegetables alongside a minimal amount of dietary fat (approximately 3–5 g per meal) is recommended, as it stimulates bile secretion and promotes micellization; in the absence of these processes, carotenoid absorption remains significantly limited even from otherwise well-processed foods [11,56]. These findings indicate that absorption efficiency is not a fixed physiological limit but can be modulated through the design of the food matrix and meal composition, providing practical, evidence-based recommendations for improving the utilization of carotenoids in the diet. Moreover, the cooking methods and timing, as well as the compound organization in the food matrix, modulate the bioavailability of carotenoids [33,57,58,59,60]. Generally, carotenoid micellization from fruits and vegetables depends on the food matrix, the carotenoid polarity, and the quantity and type of dietary fats co-ingested [6,41]. For instance, dietary fats increase the transfer of carotenoids to the aqueous micellar fraction during digestion, increasing their bioavailability [50,61]. Conversely, phytic acid, a molecule present in vegetables, reduces carotenoid bioaccessibility. To be absorbed at the enterocyte level, carotenoids, like all lipophilic compounds, must be incorporated into micelles, which are formed through the combination of bile salts, phospholipids, free fatty acids, and monoglycerides from bile and pancreatic juices [62]. Intestinal absorption of micelles enriched in carotenoids through the apical membrane is mediated by various proteins, including human class B scavenger receptor proteins (SR-B1, SR-B2, and CD36S) and NPC1L1 [41,63]. It is not clear, however, whether these proteins are actually involved in the direct transfer of carotenoids from the extracellular space into the cell or whether they are components of membrane complexes responsible for carotenoid internalization [64]. Provitamin A carotenoids such as β-carotene, α-carotene, and β-cryptoxanthin partly undergo cleavage to produce retinol and other apocarotenoids by β-carotene-oxygenase (BCO)-1 and BCO2 enzymes at the enterocyte level and in other tissues [37]. In particular, provitamin A carotenoids are enzymatically cleaved by BCO1 into retinaldehyde, which is then reduced to retinol and subsequently esterified into retinyl esters (RE). RE, together with other dietary lipids, are packaged into chylomicrons, transported into the lymphatic system and subsequently delivered to the blood. Non-provitamin A carotenoids are incorporated into chylomicrons or directed to mitochondria, where β-carotene-oxygenase-2 (BCO2) converts them into apocarotenoids [8]. Carotenoids circulate in the blood associated with LDL or HDL, and they are stored in specific carotenoid tissues [8,65]. Ninety-five percent of total carotenoids present in the blood (specifically in serum) comprise β-carotene, α-carotene, β-cryptoxanthin, lutein, zeaxanthin, and lycopene [31]. The carotenoid type influences the tissue in which it is stored. For example, lycopene is deposited mainly at the hepatic level [66]; vitamin A (retinol) is converted into a storage form (esters), loaded onto lipoproteins (chylomicrons) and accumulated in the liver [67]; lutein, zeaxanthin, and meso-zeaxanthin are deposited in the macula of the retina and in the brain [37,68]. In addition, lutein can also accumulate at the adipose tissue level [69]; β-carotene is instead stored in the liver [70].

### 4.5. Determinants of Interindividual Variability

The bioavailability and tissue concentrations of carotenoids show wide interindividual variability, which depends not only on the dietary matrix but also on host-specific factors such as age, sex, nutritional status, body fat, smoking, alcohol consumption, disease status, and medication use [71,72]. Genetic variability has also been associated with differences in carotenoid absorption and metabolism [71]. Among the most extensively studied genes are those encoding transporters such as SR-BI, CD36, and NPC1L1; cleavage enzymes such as BCO1/BCO2; and proteins involved in chylomicron secretion and peripheral distribution, such as APOB, MTTP, APOE, LDLR, GSTP1, and StARD3 [15,71]. In addition to these, there are emerging factors, in particular the gut microbiota, that may influence the bioavailability of carotenoids by altering the luminal environment and bile acid metabolism, although the evidence is still preliminary [10]. Finally, recent studies in Mediterranean populations indicate that objective biomarkers such as skin carotenoids are associated with adherence to the MD, fruit and vegetable intake, BMI, sex, age, and smoking, confirming that the response to carotenoids stems from the interaction between dietary exposure and individual characteristics [73,74,75]. From this perspective, carotenoids might represent an example of the development of precision nutrition strategies within the Mediterranean dietary pattern [71,72].

## 5. Biomarkers of Exposure and Biological Response

### 5.1. Plasma, Skin and Retinal Carotenoid Biomarkers

Various methods have been developed to evaluate the carotenoid levels in the human body, including plasma, skin and eyes. Traditionally, carotenoid levels have been measured in blood, which represents the most reliable and precise technique. The evaluation of plasma carotenoids is based on the HPLC method, coupled with UV-Vis, diode array or with the MS/MS detection [5,76,77]. The main carotenoids detected in human plasma include α-carotene, β-carotene, β-cryptoxanthin, lycopene, lutein and zeaxanthin, which are supplied by typical Mediterranean plant foods such as tomatoes and tomato products, leafy green vegetables, carrots, peppers, citrus fruits and seasonal fruits. These techniques are useful because they allow the obtaining of data for large-scale investigation purposes on the biological role of carotenoids [78,79]. However, the assessment of plasma carotenoid levels represents an expensive and invasive technique. Among the most innovative methodologies are non-invasive procedures that are based on Resonance Raman Spectroscopy (RRS), which allows the evaluation of the concentration of specific cutaneous carotenoids based on their structure [80]. RRS exploits the characteristic vibrational frequencies of the conjugated carbon–carbon double-bond system of carotenoids, generating a specific Raman signal proportional to their concentration in the skin. This approach enables rapid, objective and repeated measurements without the need for blood sampling or laboratory processing. An alternative and more economical technique, although not as reliable as RRS, is the Reflection Spectroscopy (RS), which is the technology used by portable devices, such as the Veggie Meter^®^. The Veggie Meter^®^, measuring the absorption of skin carotenoids, including α-carotene, β-carotene, β-cryptoxanthin, lycopene, and lutein and zeaxanthin, through reflection under topical pressure application, provides a numerical value expressed as “skin carotenoid score” ranging from 0 to 800 to establish the skin carotenoid level [80,81,82]. Indeed, participants gently press their finger against the convex surface of the lens with the help of a spring-loaded cover, which momentarily displaces blood from the illuminated tissue volume, reducing the influence of oxyhemoglobin and other chromophores on reflection spectra. The finger is illuminated with broadband white LED light covering the spectral range from 350 to 850 nm, and the spectral composition of diffusively reflected light is analyzed in real time. A portable computer connected to the Veggie Meter^®^ regulates light exposure, data acquisition, processing, and display of reflection spectra [83]. Interestingly, skin carotenoid levels have been found to be associated with plasma carotenoid concentrations in different age groups, including children [84,85,86,87]. However, several anthropometric and lifestyle parameters can modulate this association, including age, sex, Body Mass Index (BMI) and smoking habit, but the results vary by cohort and method, making it difficult to draw solid conclusions. The association is stronger for some carotenoids, including β-carotene, α-carotene, lutein and zeaxanthin, whereas it is weak for lycopene. Different explanations have been proposed to justify the low association between skin carotenoids and lycopene. First of all, lycopene has a trophism for skin where it preferentially accumulates more than in plasma. However, in the skin exposed to UV radiation, it rapidly undergoes oxidation or degradation, influencing skin detection. Moreover, bias by the Veggie Meter^®^ might further explain the low correlation between lycopene and skin carotenoids [88]. Another non-invasive approach to evaluate carotenoid status is represented by the assessment of ocular carotenoids, particularly those accumulated in the central retina. The xanthophyll carotenoids lutein, zeaxanthin and meso-zeaxanthin are able to selectively accumulate in the macula giving the yellow coloration of this region [89]. Unlike plasma and skin carotenoids, which provide information on systemic carotenoid exposure or peripheral tissue deposition, macular pigment optical density (MPOD) reflects the tissue-specific accumulation of retinal xanthophylls and is therefore particularly relevant as a biomarker of ocular carotenoid status. It has been reported that MPOD is influenced by dietary intake of carotenoids. In particular, it has been found that lutein and zeaxanthin supplementation can increase both circulating xanthophyll concentrations and macular pigment density [90]. However, several factors including baseline macular pigment levels, habitual dietary intake, intestinal absorption, lipid transport, genetic background and the presence of specific retinal carotenoid-binding proteins, such as glutathione S-transferase P1 for zeaxanthin and StARD3 for lutein, influence the MPOD, impacting on the objective evaluation of total carotenoids intake [91,92]. Therefore, MPOD should be considered as a functional and tissue-specific biomarker of retinal xanthophyll accumulation. MPOD is evaluated by heterochromatic flicker photometry (HFP), a non-invasive psychophysical technique based on the differential absorption of blue light by macular pigment. This technique is particularly useful, but it is strongly influenced by the participant’s visual response and operator-dependent factors. Thus, more objective techniques have also been developed to measure MPOD, including fundus reflectometry, fundus autofluorescence and RRS, which require more specialized instrumentation and are less feasible for large-scale field studies [93,94]. RRS, in particular, has been used to detect macular carotenoids on the basis of their characteristic vibrational signal, supporting its application as an objective method for assessing retinal carotenoid status [94].

### 5.2. Carotenoids as Biomarkers of Fruit and Vegetable Intake

Several studies have highlighted that plasma and skin carotenoid levels might serve as reliable, objective biomarkers reflecting short-to-medium and long-term intake of fruit and vegetables. However, the strength of the association varies based on the carotenoid, the dietary assessment and interpersonal variability. Al-Delaimy et al. have analyzed a subsample of the European Prospective Investigation into Cancer and Nutrition (EPIC) study subjects, demonstrating that food questionnaires are more accurate in predicting plasma carotenoid levels at the individual level than 24 h-recall questionnaires. Moreover, they found that plasma β-cryptoxanthin, α-carotene and lycopene can be used as a biomarker of average citrus fruit and total fruit, carrots and tomatoes intakes, respectively [95]. Among individual compounds, α-carotene showed the strongest correlations with both vegetable and fruit intake, whereas food-specific associations were observed for β-carotene-rich foods and lutein-rich foods [96]. Other authors have found that only plasma β-cryptoxanthin, lutein and zeaxanthin levels are positively associated with fruit and vegetable intake, whereas α-carotene and lycopene are negatively associated with fruit and vegetable consumption. Gender-related differences were found in the individual carotenoid levels for any given number of fruit and vegetable servings. In particular, women had higher circulating lutein levels than men [97]. Plasma carotenoids can also be useful to assess long-term high vegetable intake for women at risk of breast cancer recurrence [98]. Recently, it has been observed that the carotenoid concentration adjusted for the plasma total cholesterol is more predictive of distinguishing between high and moderate fruit and vegetable consumers [49]. Similarly, to plasma carotenoid concentration, skin carotenoid levels have been investigated as objective markers of fruit and vegetable intake. Interestingly, different studies have reported that skin carotenoids are a reliable marker of fruit and vegetable intake in children, allowing their assessment in school-based studies. In this context, we have previously demonstrated that the carotenoid score measured by Veggie Meter^®^ was higher in children attending lunch at the school compared to those eating at home [99]. Moreover, in a cohort of young adults, we observed that the carotenoid score was higher in consumers of fruits and vegetables from the garden than in those from the market, suggesting that not only the quantity, but also the quality of fresh food matters [73].

### 5.3. Carotenoids as Biomarkers of Mediterranean Diet Adherence

Although skin carotenoids are not considered a specific biomarker of adherence to the MD, a positive association has recently been found between the carotenoid score evaluated by Veggie Meter^®^ and the validated food questionnaires for the assessment of the adherence to the MD pattern, including Mediterranean Diet Adherence Screener (MEDAS) and Mediterranean Lifestyle Index (MEDLIFE). The association between the skin carotenoid score and the MD questionnaires is particularly relevant because it allows the overcoming of recall bias of the self-reported questionnaires. Indeed, response bias, arising from several factors including misunderstanding of the target concept, inaccurate recall, and social desirability, [75] may influence the accuracy of self-reported dietary intake and lead to either overestimation or underestimation of adherence to healthy dietary patterns. In this context, the objective assessment of skin carotenoid status may complement questionnaire-based approaches by providing an independent measure of carotenoid-rich fruit and vegetable exposure, thereby strengthening the overall evaluation of dietary habits.

Our research group found that the carotenoid score was associated with the Mediterranean Diet Quality Index for Children and Adolescents (KIDMED) and the MD pyramid in a cohort of adolescents [100]. Moreover, in a multiple regression model, we observed that skin carotenoid score was positively associated with KIDMED and negatively associated with waist circumference and triglyceride levels, highlighting the potential application of skin carotenoid score as a complementary biomarker for the early identification of metabolic risk in the young population [74]. In adults from Southern Italy and the Dominican Republic, skin carotenoid scores were higher in the Italian population and positively correlated with MEDAS score, suggesting that this biomarker may help capture differences in adherence to a Mediterranean dietary pattern rich in vegetables and fruit [101].

Interestingly, in the InCHIANTI cohort, a dietary biomarker-based MD score was developed including six plasma carotenoids (α-carotene, β-carotene, β-cryptoxanthin, lutein, zeaxanthin, and lycopene), selenium, linoleic acid, eicosapentaenoic (EPA) and docosahexaenoic acids (DHA), MUFAs and nine key components of the MD. The authors found that this score was inversely associated with all-cause and cardiovascular mortality in older adults [102]. Similarly, Sobiecki et al. developed a nutritional biomarker score combining five circulating carotenoids (α-carotene, β-carotene, β-cryptoxanthin, lutein, and the sum of lutein and zeaxanthin) and fatty acids, showing an inverse association with incident type 2 diabetes in EPIC-InterAct [103].

Overall, these findings highlight the potential of carotenoids as complementary tools for assessing adherence to the MD. In particular, skin carotenoid measurements offer a rapid, non-invasive, and practical approach for population-based studies and screening purposes, improving the evaluation of dietary patterns, reducing measurement error inherent to questionnaire-based methods, strengthening the investigation of associations between MD adherence and health outcomes.

## 6. Biological Activities and Mechanistic Evidence of Carotenoids

**Figure 3:**
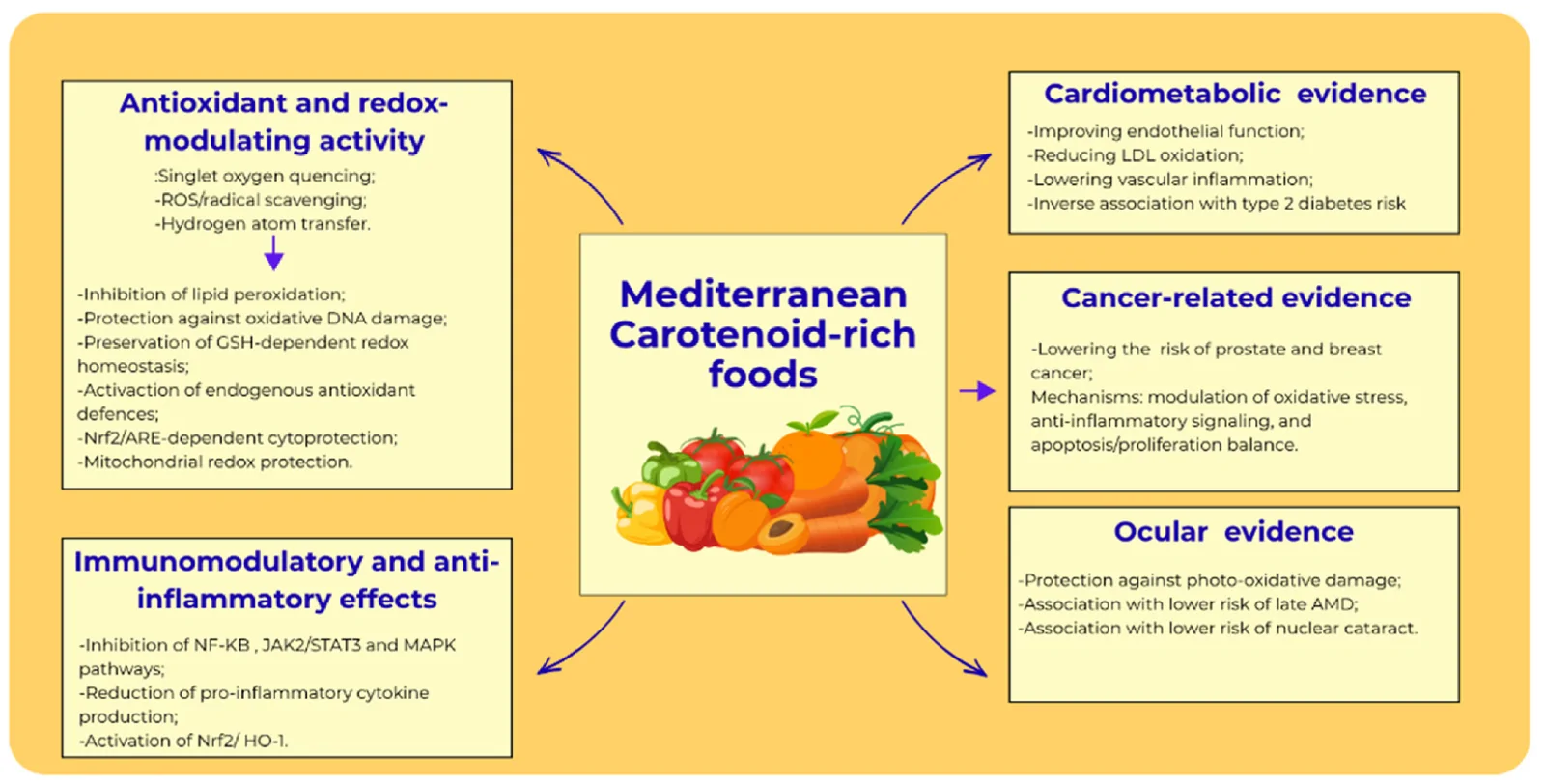
Biological activities of carotenoids in the Mediterranean Diet foods. AMD: Age-related Macular Degeneration; ARE: Antioxidant Response Element; GSH: Glutathione; HO-1: Heme oxygenase-1; JAK2: Janus Kinase 2; LDL: Low-Density Lipoprotein; NF-κB: Nuclear factor kappa-light-chain-enhancer of activated B cells; Nrf2/HO-1: Nuclear factor erythroid 2-related factor 2/Heme oxygenase-1; STAT3: Signal Transducer and Activator of Transcription 3.

Several Mediterranean foods are well studied for their healthy properties due to the presence of biomolecules, which exert different positive effects in humans. Carotenoids have been widely studied for their beneficial effects, spanning from the antioxidant to the immunomodulatory and anti-inflammatory properties (Figure 3). However, the strength of the available evidence varies substantially according to the experimental model. For instance, mechanistic findings derived from cell and animal studies should be considered supportive rather than conclusive, while human observational and intervention studies provide complementary evidence but should also be interpreted according to study design and the distinction between dietary exposure, circulating carotenoid status, and supplementation. This section summarizes the health effects of carotenoids as components of the MD pattern, highlighting their role in counteracting the development of several chronic diseases.

### 6.1. Antioxidant and Redox-Modulating Activity

Experimental evidence has reported antioxidant properties of several carotenoid-rich foods commonly consumed within the MD. Tomato, spinach, red pepper, carrot and pumpkin have been investigated for the modulatory effects on markers of oxidative stress through multiple molecular mechanisms, including direct scavenging of reactive oxygen species (ROS), inhibition of lipid peroxidation, protection against oxidative DNA damage, preservation of mitochondrial function and activation of endogenous antioxidant defence systems [47,102,104,105,106,107,108,109]. These findings provide mechanistic hypotheses that may support the importance of consuming carotenoid-rich Mediterranean plant foods as relevant dietary sources of antioxidant activity (Table 2).

Three different mechanisms by which carotenoids exert this action have been described, including the singlet oxygen quenching, hydrogen transfer and electron transfer [48]. Carotenoids quench singlet molecular oxygen (^1^O_2_) produced during the biological processes by accepting its excitation energy, thereby converting it to ground-state triplet oxygen (^3^O_2_), while the carotenoid itself is transiently converted to a triplet excited state. Subsequently, the carotenoid dissipates its energy by interaction with the surrounding solvent, returning to ground state instead of further propagating the chemical reaction. Among carotenoids, xanthophylls and carotenes, including *β*-carotene, zeaxanthin, cryptoxanthin and α-carotene, are the most efficient quenchers of ^1^O_2_. However, lycopene, mainly found in tomatoes, has been described as the most efficient biological carotenoid ^1^O_2_ quencher [117]. Carotenoids are also well known for scavenging peroxyl radicals, which form in the organism during the process of lipid peroxidation. In this case, carotenoids deactivate the peroxyl radical forming a radical adduct stabilized by resonance. Due to this property, carotenoids can protect cellular membranes and lipoproteins against oxidative damage [110]. Carotenoids can trap different radicals besides oxygen. For instance, lutein is able to scavenge sulfur radicals [111], whereas β-carotene quenches glutathione, sulfonyl, and nitrogen dioxide radicals [112]. Beyond direct antioxidant activity, carotenoids may influence redox-sensitive signaling pathways, including the Nrf2-dependent cytoprotective pathways [116].

Lipophilic extracts obtained from both fresh and processed tomato fruits were reported to protect cultured cells against oxidative stress-induced injury, indicating that tomato carotenoids may retain biological antioxidant activity even after processing. In particular, pretreatment with tomato extracts significantly reduced sodium arsenite-induced ROS accumulation, prevented intracellular glutathione depletion and completely counteracted the increase in lipid peroxidation observed under oxidative stress conditions. Interestingly, thermal processing did not significantly affect either total carotenoid content or lipophilic antioxidant activity, and extracts from processed tomatoes retained a protective efficacy comparable to that of fresh fruits [104]. Moreover, it has been found that tomato extract and its two main carotenoids, lutein and lycopene, exerted antioxidant effects modulating the levels of ROS, superoxide dismutase (SOD) and glutathione peroxidase (GPX) in H9c2 cells at a lower concentration range (0.5–5 μM), whereas higher nominal concentrations were associated with a loss of antioxidant efficacy and pro-oxidant responses. The loss of the antioxidant activity of these molecules might be due to the poor solubility of carotenoids, which facilitates their precipitation in cell culture media [102].

Although one of the most studied foods from the MD is tomato, other products have been studied for their antioxidant activity. Arru and collaborators investigated the antioxidant activity of different *Spinacia oleracea* extracts and of the main components of its phytocomplex, including lutein and β-carotene, in human HT29 colorectal cells exposed to oxidative stress, reporting that both carotenoids protected cells against H_2_O_2_-induced oxidative DNA damage and were also able to counteract the increase in intracellular ROS induced by menadione [47]. Another important Mediterranean source of an antioxidant carotenoid is red pepper, particularly enriched in capsanthin and capsorubin. These xanthophylls have been shown to protect human dermal fibroblasts against ultraviolet B-induced oxidative DNA damage, reducing DNA strand breaks after oxidative challenge in vitro [105]. Carrot-derived carotenoids have similarly demonstrated antioxidant activity in biological models [106]. Interestingly, pumpkin carotenoid extracts attenuated oxidative stress and mitochondrial dysfunction in a rat model of doxorubicin-induced cardiotoxicity [109].

Importantly, the magnitude of this antioxidant activity appears to depend not only on the concentration of individual carotenoids but also on the composition of the whole food phytocomplex. This suggests that carotenoids may act synergistically with other phytochemicals, including polyphenols, vitamins and additional redox-active compounds naturally present in plant foods. For instance, β-carotene interacts with vitamins C and E, exerting a synergistic response [110]. Moreover, it has been reported that also the culinary process influenced carotenoid release from the matrix and their absorption. A small, randomized crossover study has demonstrated that the addition of olive oil to tomato juice increased the postprandial appearance of lycopene and other carotenoids along with a more favourable postprandial lipid response than tomato juice alone. In particular, all lycopene isomers increased significantly, whereas LDL cholesterol and total cholesterol decreased significantly in subjects consuming tomato juice with oil compared to the control group [42]. Similarly, it has been found that the consumption of processed tomato products with a meal attenuated postprandial lipemia-associated oxidative stress and reduced high-fat meal-induced LDL oxidation in healthy adults [113]. Recently, tomato powder increased total antioxidant capacity and reduced the levels of biomarkers of lipid peroxidation in well-trained athletes. However, the authors have demonstrated that this effect is dependent not only on lycopene but involves its interaction with bioactive components [114].

Green leafy vegetables represent sources of lutein and zeaxanthin, but their effects on systemic oxidative-stress markers are not well established. Indeed, in healthy volunteers, ten days of a spinach preparation significantly increased plasma lutein and produced a tendency toward lower indices of lipid peroxidation, while total plasma antioxidant capacity did not change [118]. Similarly, in a separate 8-week randomized food intervention, spinach powder and carrot juice increased the relevant circulating carotenoids but did not significantly modify oxidation in healthy volunteers [119]. In a crossover intervention involving healthy women, the consumption of blood orange juice for 21 days improved the cellular stress-response, without generalized reduction of oxidative biomarkers. Indeed, it has been found that this drink increased plasma vitamin C, cyanidin-3-glucoside, beta-cryptoxanthin and zeaxanthin and improved lymphocyte resistance to oxidative DNA damage. However, plasma antioxidant capacity, malondialdehyde and urinary 11-dehydro-thromboxane B2 were unchanged [115]. Interestingly, a study conducting a systematic review and a meta-analysis of controlled carotenoid interventions has found significantly higher levels of antioxidative parameters, including ferric-reducing ability of plasma (FRAP) and oxygen radical absorbance capacity (ORAC) and decreased blood triglyceride level. However, no differences were found in antioxidant enzyme levels or in other lipid and lipoprotein parameters between the subjects treated with carotenoids and the control group [120]. The antioxidant activity of xanthophylls has also been investigated in human tissues under conditions of experimentally induced oxidative stress. In a randomized double-blind study, oral and/or topical administration of lutein and zeaxanthin significantly reduced UV-induced skin lipid peroxidation, assessed by malondialdehyde levels, compared with placebo [121].

### 6.2. Immunomodulatory and Anti-Inflammatory Effects

Besides their antioxidant properties, carotenoids have been investigated for their ability to modulate immune and inflammatory responses through both redox-dependent and immune-specific mechanisms.

Experimental studies suggested that the anti-inflammatory activity of carotenoids might be associated with the modulation of redox-sensitive and inflammatory signaling pathways, including the NF-κB signaling pathway. For instance, β-carotene was reported to reduce LPS-induced production of nitric oxide, prostaglandin E2, TNF-α and IL-1β in RAW 264.7 and primary macrophages by inhibiting IκBα phosphorylation and degradation, preventing nuclear translocation of the NF-κB p65 subunit and its activation [122]. Moreover, it has been demonstrated that the anti-inflammatory effects of β-carotene as well as lycopene are associated with the activation of other pathways besides the NF-κB pathway, including JAK2/STAT3 and JNK/p38 MAPK signaling [123,124]. Interestingly, concentrations of lycopene that exerted anti-inflammatory effects in LPS-stimulated RAW 264.7 also resulted in decreased macrophage migration and reduced inflammatory signaling in adipocytes exposed to macrophage-conditioned medium [125]. Moreover, in TNF-α-stimulated endothelial cells, lycopene was associated with the activation of the Nrf2/HO-1 antioxidant pathway, increased intracellular glutathione and reduced IκB degradation, NF-κB nuclear translocation and ICAM-1 expression, suggesting a possible crosstalk between antioxidant defence and suppression of inflammatory signaling [126]. In a randomized crossover trial involving subjects at high cardiovascular risk, daily consumption of tomato juice for four weeks significantly reduced circulating ICAM-1 and VCAM-1 concentrations, which was associated with circulating trans-lycopene concentrations, suggesting a contribution of tomato carotenoids to the modulation of vascular inflammation [127]. Consistent with these observations, consumption of a traditional tomato-based sofrito also influenced circulating inflammatory biomarkers. In healthy men, a single dose of sofrito significantly reduced plasma TNF-α concentrations, although no association was found with lycopene [46]. A randomized crossover trial investigated the effect of adding 100 g of tomato puree to a high-fat meal in healthy men, demonstrating that tomato puree significantly attenuated the postprandial inflammatory response, reducing plasma TNF-α and IL-6 and decreasing the expression of TNFA, IL6, IL1B and CCL5 in adipose tissue compared with the high-fat meal alone. However, it has not been evaluated whether these effects were associated with carotenoid content [128]. In LPS-stimulated BV-2 microglial cells, lutein treatment was associated with reductions in several inflammatory mediators and changes in redox-sensitive signaling pathways. Indeed, lutein significantly reduced iNOS and COX-2 expression and TNF-α, IL-1β and nitric oxide production in LPS-stimulated BV-2 microglia. Preliminary studies, which require further validation, suggest that lutein inhibited NF-κB activation by suppressing p38, JNK and Akt phosphorylation and, at the same time, enhanced ERK1/2-dependent Nrf2 activation and the expression of antioxidant proteins, including HO-1 and NQO1 [129]. Lutein may also exert anti-inflammatory effects through modulation of intracellular cytokine signaling. In endotoxin-induced retinal inflammation, lutein reduced STAT3 activation and prevented pathological glial fibrillary acidic protein upregulation in Müller glial cells, concomitantly with a reduction in oxidative stress and preservation of photoreceptor function [130].

### 6.3. Carotenoids, Gut Microbiota and Intestinal Immune Homeostasis: Emerging Evidence

An emerging area of research is focused on the interaction between dietary carotenoids and gut microbiota. Indeed, a proportion of dietary carotenoids is not absorbed in the small intestine and reaches the colon, where interactions with the gut microbiota may generate carotenoid-derived metabolites characterized by biological activity. Several mechanisms through which carotenoids may influence gut microbiota, promoting the abundance of healthy bacteria, including *Bifidobacterium* and *Lactobacillus*, have been proposed [51,131]. For instance, β-carotene and lycopene were associated with reduced intestinal NF-κB activation and pro-inflammatory cytokine production, along with improved expression of tight-junction proteins such as occludin and claudin-1. In addition, carotenoids and their metabolites may further interact with nuclear receptors and transcription factors, including PPARγ, RAR/RXR and NF-κB, thereby modulating microbial homeostasis and immune regulation [51]. The food matrix may further influence the interaction between carotenoids and the intestinal microbiota. For instance, raw and boiled tomatoes retained a greater carotenoid fraction available for the subsequent colonic fermentation phase, promoting the growth of beneficial microbiota while reducing the ratio of *Firmicutes/Bacteroides* [132]. However, these results were obtained using in vitro simulated gastrointestinal digestion and colonic fermentation and therefore require confirmation in vivo. Human evidence is still limited. In a small cross-sectional study including 23 pregnant women with complete dietary and microbiota data, consumption of foods rich in provitamin A carotenoids was associated with higher fecal bacterial α-diversity in pregnant women. Moreover, plasma α-carotene and β-carotene concentrations were also positively associated with several measures of microbial diversity [133]. However, due to dietary factors including fiber intake, it was not possible to understand whether the microbial diversity was associated with a specific effect of carotenoids rather than the overall dietary pattern [133]. More recently, Valdes et al. investigated the relationship between gut metagenome composition and vitamin A-related metabolites and carotenoid metabolites. Five vitamin A-related serum metabolites were positively correlated with microbiome alpha diversity, whereas carotenoid compounds were positively associated with the short-chain fatty-acid-producing bacteria *Faecalibacterium prausnitzii* and *Coprococcus eutactus*. Moreover, the healthy eating index (HEI) was strongly associated with gut microbiome diversity and with all carotenoid compounds [134]. Recent ex vivo evidence further suggests that the effects of carotenoids on the gut microbiota may depend on the dietary context and on the characteristics of the host microbiome. Using fecal microbiota from adults with irritable bowel syndrome or overweight, it has been found that β-carotene, lutein, lycopene and zeaxanthin combined with the prebiotic fiber inulin induced more pronounced changes in microbial composition and enhanced SCFA production, particularly butyrate [135]. This aspect is particularly relevant to the MD pattern, in which carotenoids are mainly consumed through fruits and vegetables, which are enriched with dietary fiber, polyphenols and unsaturated lipids. Taken together, the available evidence suggests that carotenoids may participate in diet–microbiota interactions, although their independent contribution remains difficult to separate from that of the whole dietary matrix. Nevertheless, a specific carotenoid-mediated link between adherence to the MD and gut microbiota composition has not yet been demonstrated.

## 7. Human Health Evidence

### 7.1. Cardiometabolic Evidence

Carotenoid intake, circulating concentrations and supplementation have been investigated in relation to cardiometabolic outcomes. Among carotenoids, lycopene has been particularly examined in relation to cardiovascular health. Lycopene may contribute to vascular protection through several mechanisms, including the attenuation of oxidative stress and inflammation and the preservation of endothelial function. In a randomized double-blind placebo-controlled trial in patients with established cardiovascular disease and healthy volunteers, supplementation with 7 mg/day lycopene for two months improved endothelium-dependent vasodilation in statin-treated patients with cardiovascular disease, whereas no significant improvement was observed in healthy volunteers, suggesting that the vascular response to lycopene may be more evident under conditions characterized by endothelial dysfunction and increased cardiovascular risk [136]. Consistent with these findings, a systematic review and meta-analysis of 21 intervention studies showed that tomato-based interventions significantly reduced LDL cholesterol and IL-6 concentrations and improved flow-mediated dilation. Moreover, lycopene supplementation was associated with a reduction in systolic blood pressure. These findings suggest that responses to tomato-based interventions may reflect the contribution of multiple components of the food matrix rather than lycopene alone [137]. However, Cheng and collaborators performed a systematic review and meta-analysis demonstrating that the highest lycopene intake or circulating concentrations are associated with a significantly lower risk of stroke, cardiovascular disease and a lower risk of mortality. Conversely, no significant association was observed with myocardial infarction, while evidence for atherosclerosis, heart failure and atrial fibrillation remained limited [137]. However, higher circulating concentrations of lutein, zeaxanthin and β-cryptoxanthin have been associated with slower progression of subclinical atherosclerosis, reducing the progression of carotid intima-media thickness [138]. Carotenoid status has also been associated with glucose metabolism and type 2 diabetes. In particular, Jiang and collaborators evaluated both dietary intake and circulating concentrations of the six major carotenoids, including α-carotene, β-carotene, β-cryptoxanthin, lycopene, lutein and zeaxanthin, in relation to incident type 2 diabetes. Higher dietary intake of total carotenoids, α-carotene, β-carotene and lutein/zeaxanthin was associated with a lower risk of disease, with the strongest association observed for β-carotene. Consistently, higher circulating concentrations of total carotenoids, β-carotene, lycopene and lutein were also inversely associated with type 2 diabetes risk. Interestingly, the associations were not fully concordant when dietary intake and circulating concentrations were compared. For instance, dietary lycopene was not significantly associated with diabetes risk, whereas higher circulating lycopene concentrations were associated with a lower risk, suggesting that different factors, including bioavailability, food processing, lipid co-consumption, and body composition, may influence circulating carotenoid levels [139].

### 7.2. Ocular Health

Ocular health represents another one of the most extensively investigated areas for carotenoid-related health outcomes. Lutein and zeaxanthin represent the two main macular pigments, which may contribute to retinal protection through both blue-light filtering and antioxidant mechanisms [140]. Overall, higher intake of lutein and zeaxanthin was not significantly associated with early age-related macular degeneration (AMD), whereas a significant inverse association was observed for late AMD. Moreover, a stronger association was observed for neovascular AMD, for which higher lutein and zeaxanthin intake was associated with a 32% lower risk [141]. Beyond AMD, the effects of lutein and zeaxanthin have also been investigated in age-related cataract, demonstrating that higher dietary intake of these two compounds was associated with a reduced risk of nuclear cataract, with an approximately 3% reduction in risk for every 300 μg/day increase in intake [142]. Interestingly, the association with nuclear cataract risk was stronger for zeaxanthin than for lutein, due to differences in their distribution and biological properties within the lens [143]. It is worth to note that, although observational studies have reported inverse associations between dietary or circulating lutein and zeaxanthin concentrations and age-related cataract, evidence from randomized controlled trials is less consistent. Indeed, supplementation with lutein and zeaxanthin for a median follow-up of 4.7 years did not significantly reduce the overall risk of cataract surgery or vision loss, whereas a lower risk of cataract surgery was observed among participants in the lowest quintile of baseline dietary lutein/zeaxanthin intake [144]. Ranard et al. concluded that the available evidence may support consideration of dietary guidance for lutein intake, although no universally accepted dietary reference intake has been established [145,146]. Lutein and zeaxanthin have also been investigated in relation to pre- and postnatal visual development, reinforcing the importance of dietary suggestion and supplementation strategies to sustain fetal and infant growth [147]. Moreover, beyond the retina, lutein is preferentially accumulated in human brain tissue and represents the predominant carotenoid in both infant and adult brains. Associations between brain or macular lutein status and cognitive performance have been reported [148,149], although much of the available evidence remains observational and does not establish causality.

### 7.3. Cancer-Related Evidence

Finally, carotenoids have also been extensively investigated in relation to cancer risk and cancer-related outcomes. Experimental studies have shown that these compounds can interfere with several processes involved in carcinogenesis, including oxidative stress, inflammatory signaling, cell proliferation, apoptosis, retinoid-dependent pathways and cell-to-cell communication. However, the research is still ongoing and contrasting data have been reported, suggesting the need to further investigate the role of carotenoids in cancer prevention. Overall, higher carotenoid exposure was associated with a lower risk of total cancer and several types of malignancies, including cancers of the digestive system, prostate, breast, head and neck, gynecologic and hematologic cancers. Importantly, while dietary intake and circulating carotenoid concentrations were generally associated with a lower cancer risk, supplementation did not reproduce these associations. In particular, previous randomized trials conducted in smokers and other high-risk populations showed that high-dose β-carotene supplementation increased lung cancer incidence rather than preventing it. The authors suggested that under conditions of elevated oxidative stress, such as cigarette smoke exposure, high concentrations of isolated β-carotene may exhibit pro-oxidant activity rather than the antioxidant effects generally associated with dietary exposure [150]. Nevertheless, subgroup analyses showed inverse associations for different individual carotenoids, including α-carotene, β-carotene, β-cryptoxanthin, lutein/zeaxanthin and lycopene [151].

An inverse association has been found between dietary intake of lycopene as well as circulating lycopene levels and risk of prostate cancer [152]. However, lycopene supplementation did not affect the levels of prostate-specific antigen (PSA) levels [152,153]. Higher circulating carotenoid concentrations have also been inversely associated with breast cancer risk, with generally stronger associations observed for plasma carotenoid levels than for dietary intake estimated by questionnaires [153,154].

### 7.4. Side Effects and Nutrient Interactions of Carotenoids

Although carotenoids showed positive effects in the prevention of several diseases, it has been found that high-dose supplementation might induce side effects. Recently, a study conducting a systematic review and a meta-analysis of eight randomized controlled trials has found that β-carotene supplementation was associated with an increased risk of lung cancer (RR = 1.16, 95% CI 1.06–1.26), with a stronger association among smokers, asbestos-exposed individuals (RR = 1.21, 95% CI 1.08–1.35) and non-medics (RR = 1.18, 95% CI = 1.07–1.29) [155]. Previously, the α-Tocopherol, β-carotene Cancer Prevention (ATBC) Study has demonstrated that β-carotene supplementation was associated with a higher incidence and mortality of lung cancer compared with no β-carotene supplementation in male smokers [156]. Similarly, other authors have found that β-carotene supplementation increased lung cancer risk among male smokers regardless of the tar or nicotine content of the cigarettes smoked [157]. These findings indicate that effects observed for carotenoid-rich foods or circulating carotenoid concentrations should not be extrapolated to high-dose isolated β-carotene supplementation, particularly in smokers. High intake of carotene-rich foods has been also associated with diet-induced carotenodermia [158]. Similarly, supplementation with lutein for 4 months was associated with the development of carotenodermia, although no concomitant hematological or biochemical abnormalities were detected [159]. Also, lycopene supplementation can result in carotenodermia [160]. Safety profiles also differ among xanthophylls. Although not one of the principal dietary xanthophylls considered in the Mediterranean dietary pattern, canthaxanthin provides a well-documented example of compound-specific carotenoid toxicity: prolonged high exposure has been associated with dose-dependent crystalline retinal deposits known as canthaxanthin retinopathy, which generally regress after discontinuation but may persist for prolonged periods and, in rare cases, be associated with visual impairment [161].

Carotenoid exposure should also be interpreted in the context of nutrient–nutrient interactions. Dietary lipids facilitate carotenoid release and incorporation into mixed micelles, with systematic evidence indicating that unsaturated fats can enhance carotenoid bioaccessibility and bioavailability [162]. Moreover, interactions among carotenoids, including those between β-carotene and lutein or canthaxanthin, may occur during digestion through competition for incorporation into mixed micelles, interference with provitamin A carotenoid cleavage, and exchange among lipoproteins after absorption [163,164]. Other dietary components, including divalent minerals, may also influence carotenoid bioaccessibility through interactions with bile acids and lipid digestion, although their clinical relevance remains less clearly established [165].

## 8. Conclusions and Future Perspectives

Carotenoids represent one class of bioactive compounds in MD foods. However, their biological relevance cannot be captured by dietary intake or circulating concentration alone. As discussed, the amount of carotenoids ultimately available for absorption is shaped at every step by food matrix effects (from the physical structure of the plant tissue to processing and co-ingested lipids), while host-related factors, including genetic variation, physiological status, body composition, and lifestyle, drive substantial interindividual variability in absorption, distribution, and tissue accumulation. More recently, the gut microbiome has emerged as a further modulator of this axis, both by acting on the fraction of carotenoids that escapes small-intestinal absorption and by mediating downstream effects on host redox and inflammatory status. Taken together, these interactions between food and the host’s microbiome make carotenoids a paradigmatic example of systemic nutrition, in which it is necessary to distinguish between bioavailability, that is, the fraction that is absorbed, and bioefficacy, that is, the extent to which absorbed carotenoids result in a measurable functional or health-related outcome. Framing carotenoids in these terms shifts their conceptual role from that of isolated dietary antioxidants to that of functional components embedded within a broader nutritional and redox network, whose biological impact is contingent on matrix, host, and microbial context rather than intake alone.

This shift has direct implications for how carotenoid-rich Mediterranean dietary patterns are positioned within preventive and personalized nutrition. Rather than being considered solely as sources of specific bioactive pigments, such patterns should be viewed as modifiable levers capable of shaping carotenoid bioaccessibility, bioefficacy, and downstream biomarker profiles in ways that are relevant to chronic disease risk reduction and healthy ageing. Realizing this potential will require moving beyond population-level recommendations toward precision nutrition approaches that address the marked interindividual variability in bioavailability and metabolic handling—particularly driven by genetic and microbiome-related determinants. In parallel, the growing feasibility of objectively assessing carotenoid exposure through circulating, cutaneous, and macular biomarkers offers a complementary, non-invasive toolkit to refine dietary assessment and adherence monitoring: while plasma levels reflect systemic exposure, rapid skin carotenoid measurements provide a reliable surrogate to complement traditional dietary questionnaires.

Finally, emerging evidence linking carotenoid exposure to microbial diversity and metabolic activity highlights the need for truly integrative frameworks. Future research should combine dietary exposure, host genetics, microbiome profiles, and objective multi-tissue biomarkers within well-characterized, longitudinal cohorts. The explicit goal must be to define host-specific carotenoid response phenotypes to translate them into tailored dietary strategies that maximize the bioefficacy, not merely the bioavailability, of carotenoids within Mediterranean and plant-rich dietary patterns.

## Figures and Tables

**Table 2:** Mechanisms and biological consequences of major carotenoid-rich Mediterranean plant foods.

Mechanism	Main Carotenoids Involved	Reported Experimental Outcome	References
Singlet oxygen quenching	Lycopene, β-carotene and xanthophylls	Prevention of ^1^O_2_-mediated oxidative reactions	[110]
Hydrogen atom transfer (HAT)	Capsanthin and other carotenoids	Termination of radical chain reactions	[106]
Free-radical scavenging and radical-adduct formation	β-carotene, lutein and other carotenoids	Limitation of radical propagation and oxidative chain reactions	[110,111,112]
Inhibition of lipid peroxidation	Lycopene, β-carotene and mixed food carotenoids	Protection of cellular membranes and lipoproteins against oxidative damage	[104,113,114]
Protection against oxidative DNA damage	Lutein, β-carotene, capsanthin and capsorubin	Reduced oxidative DNA damage and preservation of genomic integrity	[47,105,115]
Preservation of glutathione-dependent redox homeostasis	Tomato carotenoids within the food phytocomplex	Maintenance of intracellular antioxidant buffering	[104]
Modulation of endogenous antioxidant defenses	Lycopene, lutein and tomato carotenoid extracts	Improved cellular control of ROS	[102]
Nrf2/ARE-dependent redox modulation	Lycopene, β-carotene and other carotenoids	Enhancement of endogenous antioxidants and phase II defense systems	[102,116]
Mitochondrial redox protection	Mixed carotenoid-rich extracts	Preservation of mitochondrial redox homeostasis and function	[108,109]

## Data Availability

All data generated or analyzed during this study are included in this article. Further enquiries can be directed to the corresponding author.
